# Endothelin-1 as a Mediator of Heme Oxygenase-1-Induced Stemness in Colorectal Cancer: Influence of p53

**DOI:** 10.3390/jpm11060509

**Published:** 2021-06-04

**Authors:** Sandra Ríos-Arrabal, Jose D. Puentes-Pardo, Sara Moreno-SanJuan, Ágata Szuba, Jorge Casado, María García-Costela, Julia Escudero-Feliu, Michela Verbeni, Carlos Cano, Cristina González-Puga, Alicia Martín-Lagos Maldonado, Ángel Carazo, Josefa León

**Affiliations:** 1 Instituto de Investigación Biosanitaria de Granada, ibs.GRANADA, 18012 Granada, Spain; sandrariosarrabal@hotmail.com (S.R.-A.); josedavidpupa@correo.ugr.es (J.D.P.-P.); sara.moreno@ibsgranada.es (S.M.-S.); jorgecasado@ugr.es (J.C.); gmaria92@correo.ugr.es (M.G.-C.); juliaescuderofeliu@gmail.com (J.E.-F.); crisgona2@hotmail.com (C.G.-P.); aliciamartin-lagos@hotmail.com (A.M.-L.M.); angelcarazogallego@gmail.com (Á.C.); 2 Departamento de Farmacología, Facultad de Farmacia, Universidad de Granada, 18071 Granada, Spain; 3 Cytometry and Microscopy Research Service, Instituto de Investigación Biosanitaria de Granada, ibs.GRANADA, 18012 Granada, Spain; 4 Unidad de Gestión Clínica de Cirugía, Complejo Hospitalario de Jaén, 23007 Jaén, Spain; agata_szuba@wp.pl; 5 Departamento de Ciencias de la Computación e Inteligencia Artificial, E.T.S. de Ingenierías Informática y de Telecomunicación, Universidad de Granada, 18014 Granada, Spain; michelav@decsai.ugr.es (M.V.); ccano@decsai.ugr.es (C.C.); 6 Unidad de Gestión Clínica de Cirugía, Hospital Universitario San Cecilio de Granada, 18016 Granada, Spain; 7 Unidad de Gestión Clínica de Aparato Digestivo, Hospital Universitario San Cecilio de Granada, 18016 Granada, Spain

**Keywords:** colorectal cancer, cancer stem cells, heme oxygenase-1, endothelin-1, endothelin con-verting enzyme-1, bosentan

## Abstract

Heme oxygenase-1 (HO-1) is an antioxidant protein implicated in tumor progression, metastasis, and resistance to therapy. Elevated HO-1 expression is associated with stemness in several types of cancer, although this aspect has not yet been studied in colorectal cancer (CRC). Using an in vitro model, we demonstrated that HO-1 overexpression regulates stemness and resistance to 5-FU treatment, regardless of p53. In samples from CRC patients, HO-1 and endothelin converting enzyme-1 (ECE-1) expression correlated significantly, and p53 had no influence on this result. Carbon monoxide (CO) activated the ECE-1/endothelin-1 (ET-1) pathway, which could account for the protumoral effects of HO-1 in p53 wild-type cells, as demonstrated after treatment with bosentan (an antagonist of both ETRA and ETRB endothelin-1 receptors). Surprisingly, in cells with a non-active p53 or a mutated p53 with gain-of-function, ECE-1-produced ET-1 acted as a protective molecule, since treatment with bosentan led to increased efficiency for spheres formation and percentage of cancer stem cells (CSCs) markers. In these cells, HO-1 could activate or inactivate certain unknown routes that could induce these contrary responses after treatment with bosentan in our cell model. However more research is warranted to confirm these results. Patients carrying tumors with a high expression of both HO-1 and ECE-1 and a non-wild-type p53 should be considered for HO-1 based-therapies instead of ET-1 antagonists-based ones.

## 1. Introduction

Worldwide, colorectal cancer (CRC) annually affects more than one million men and women and causes more than half a million deaths [1]. Drug-resistance remains one of the challenges for the low survival rates of patients in advanced stages of the disease [2].

Tumor heterogeneity associated with changes in gene expression or in epigenetics supports the existence of therapy-resistant cancer cells. These cells, usually called cancer stem cells (CSCs) or tumor-initiating cells (TICs), represent a small fraction within the cancer and are also responsible for initiating, maintaining, and developing cancer growth [3]. Therefore, current research has been focused on the discovery of molecular targets involved in the appearance and maintenance of CSCs [3].

The enzyme heme oxygenase-1 (HO-1) or heat shock protein 32 (Hsp32) is the inducible isoform of HO, which catalyzes the limiting step in the oxidation of the heme group in equimolar quantities of CO, ferrous iron, and biliverdin [4]. HO-1 is a protein sensitive to oxidative stress, growth factors, pathogen-associated molecular patterns (PAMPs), cytokines, metalloporphyrins, heme, and heavy metals. It has been widely accepted that HO-1 is a cytoprotective protein in several pathological conditions, acting as an anti-inflammatory, antioxidant, and antiapoptotic agent through several mechanisms [5,6,7,8]. On the contrary, in some cell types and under certain circumstances, it may amplify intracellular oxidative stress and exacerbate the disease process [9].

Overexpression of HO-1 has been observed in preneoplastic and neoplastic tissues [4]. It has been implicated in tumor progression, invasiveness, angiogenesis, metastasis, and immune scape, acting at both microenvironment and tumor levels [10,11]. These effects are highly dependent on the intracellular localization of HO-1 (nuclear, mitochondrial, or cytoplasmic) [12,13] and the type of cancer studied [10]. Recent reports showed that at low levels of expression, HO-1 induces cancer progression, while excessive activation of HO-1 leads to cell death by activating ferroptosis [14], which could explain the contradictory effects found in several types of cancer, including CRC [10]. Specifically, in this type of cancer, HO-1 overexpression is associated with long-term survival in patients [15], reducing cell viability through induction of cell cycle arrest and apoptosis in tumor cells, although a functional p53 protein is required for these effects [16]. However, and contrary to this, other reports showed that overexpression of HO-1 mediates EGF-induced colon cancer cell proliferation [17] and promotes tumor progression and metastasis of colorectal carcinoma cells by inhibiting antitumor immunity [18] and increasing angiogenesis [19,20].

HO-1 has also been implicated in therapy resistance in a variety of cancers, including CRC [21,22]. As mentioned before, this ability of cancers to escape therapy has been attributed to the existence of subpopulations of CSCs, which activate DNA repair, increase drug efflux, and their quiescent state [23]. Elevated HO-1 expression is associated with stemness and cell self-renewal in glioblastoma, [24], breast cancer [25], and in leukemia [26]. However, this aspect remains to be studied in CRC.

Endothelins (ETs) are a family of three 21 amino-acid peptides (ET-1, 2 and 3). Prepro-ET-1 peptide is the primary translation product of the ET-1 gene (EDN-1), which is finally cleaved by an endothelin-converting enzyme (ECE-1) to form the biologically active ET-1. This peptide mediates physiological functions such as vasoconstriction and cell proliferation in vascular and non-vascular tissues through two cell-surface receptors, ETRA and ETRB [27,28]. In tumor cells, ET-1 promotes angiogenesis in addition to cell proliferation, metastasis, and suppression of apoptosis [28]. It was proposed that CSCs from CRC secrete ET-1 and that the activation of the ETRA receptor/β-arrestin1 axis induces the cross-talk with β-catenin signaling to sustain stemness, the epithelial-to-mesenchymal transition (EMT) phenotype, and regulates the response to chemotherapy [29].

Previous studies unrelated to cancer have reported a relationship between HO-1 and the ECE-1/ET-1 pathway. HO-1-produced CO regulates ET-1 production by smooth muscle cells under normal and hypoxic conditions [30] and in several models of disease [31,32,33]. In addition, chemical inhibition of ECE-1 lead to HO-1 overexpression on ischemic-reperfusion spinal cord injury in rats [34].

Taking into account all the above, in this study, we aimed to investigate the role of HO-1 in regulating stemness in CRC and the mediating effects of the ECE-1/ET-1 system in this process.

## 2. Materials and Methods

### 2.1. Cell Culture and Reagents

Three CRC cell lines with different p53 statuses were used in this study: HCT-116 (p53 wild-type), HCT-116 p53 -/- (p53 null), and HT-29 (p53 mutated). HCT-116 and HCT-116 p53 null were obtained from Horizon Discovery (Cambridge, UK). HT-29 cells were obtained from American Type Culture Collection (ATCC, Rockville, MD, USA). All cell types were cultivated in RPMI-1640 medium (Gibco, Carlsbad, CA, USA) supplemented with 2 mM L-glutamine, 10% FBS, and a 1% antibiotic-antimycotic cocktail containing penicillin (100 U/mL), streptomycin (100 μg/mL), and amphotericin B (250 ng/mL) (Gibco, Carlsbad, CA, USA) at 37 °C with 5% CO_2_.

CORM3, biliverdin, hemin, and CoPPIX were purchased from Sigma-Aldric Co. (St. Luis, MO, USA). Bosentan was purchased from Selleckchem (Houston, TX, USA).

### 2.2. Patients

This research work was part of a larger, ongoing prospective study that aimed to investigate CSCs regulatory pathways in CRC. Initially, 183 patients were recruited, of which 150 were included in this study [35]. Samples were obtained from patients who underwent surgery for primary sporadic CRC and were provided by the Andalusian Tumor Bank Network (RBTA) (Table S1). Viable tumor tissues and adjacent normal mucosa were dissected immediately and fresh-frozen in Tissue-Tek1 (Optimal Cutting Temperature Compound, Sakura Finetek Europe B.V., Zoeterwoude, The Netherlands). The inclusion criteria included people over 18 years, without hereditary burden, not treated with neoadjuvant therapy, and not previously diagnosed or treated for cancer. The Ethical Committee of Clinical Research of Granada (project code: PI-067/2013; date of approval: 24 January 2014) approved the study. All patients gave written informed consent for the use of samples in biomedical research.

### 2.3. Transient Transfection of HO-1

Cells at 60–70% of confluence were transiently transfected with lipofectamine 2000 transfection reagent (Thermo Fisher Scientific, Waltham, MA, USA) according to the manufacturer’s instructions. Transfected cells were then collected 24–96 h after transfection. Plasmid expression vector pCMV6 containing the human *HMOX1* gene and the corresponding empty vector were purchased from Origene Technologies (Rockville, MA, USA).

### 2.4. Western Blotting

After treatments, cells were washed in ice-cold DPBS and incubated in RIPA buffer containing protease inhibitors. Then, proteins were separated by SDS-PAGE and transferred to PVDF filters. Finally, blots were probed with appropriate antibodies raised against HO-1 (Abcam, Cambridge, UK), ECE-1 (Abcam, Cambridge, UK), NF-κB (p65; Santa Cruz Biotechnology, Inc., Dallas, TX, USA), pNF-κB at Ser^529^ (phospho-p65; Becton Dickinson, Franklin Lakes, NJ, USA), AP-1 (c-jun; Santa Cruz Biotechnology, Inc., Dallas, TX, USA), pAP-1 at Ser^243^ (phosphor-c-jun; Santa Cruz Biotechnology, Inc., Dallas, TX, USA), and β-actin (Santa Cruz Biotechnology, Inc., Dallas, TX, USA). Proteins were visualized by enhanced chemiluminescence using appropriate HRP-conjugated secondary antibodies (Santa Cruz Biotechnology, Inc., Dallas, TX, USA). The intensity of the bands was estimated using Quantity One 4.6.8 (Bio-Rad Laboratories, Inc., Hercules, CA, USA) for Windows analytic software.

### 2.5. ET-1 ELISA

ET-1 was analyzed in 100 μL of conditioned media using a colorimetric ELISA kit (R & D Systems, Minneapolis, MN, USA) in accordance with the manufacturer’s protocol using a microplate reader TRIAD Multimode Reader series (Dynex Technologies, Chantilly, VA, USA) at 450 nm. Results were determined by comparison with standard curves and normalized to the number of cells in each well.

### 2.6. RNA Isolation and cDNA Synthesis

Total RNA from tissue samples was isolated using the RNeasy Mini Kit (Qiagen, Hilden, Germany). The amount of total RNA was determined by UV spectrophotometry, and RNA integrity was assessed by agarose gel electrophoresis. First-strand cDNA was prepared by a reverse transcription cDNA synthesis kit (qScript cDNA Synthesis, Quantabio, Beverly, MA, USA).

### 2.7. Real Time PCR

Quantitative analysis of mRNA expression was performed using the PerfeCTa SYBR Green SuperMix Kit (Quantabio, Beverly, MA, USA) on a CFX96 Dx Real-Time PCR Detection System (Bio-Rad, Hercules, CA, USA) following manufacturer’s instructions. Expression of mRNA was evaluated through standard curves generated for each target gene by plotting Ct values versus log cDNA dilution. UBC was used to normalize mRNA levels. PCR products were verified by a melting profile and agarose gel electrophoresis to rule out nonspecific PCR products and primer dimers.

### 2.8. DNA Extraction and p53 Mutations Analysis

DNA was extracted from tissues using the QIAamp ADN mini Kit (Qiagen, Hilden, Germany) and quantified on a NanoDrop ND-1000 spectrophotometer (Implen GmbH, Munich, Germany). Mutations were analyzed in 2–10 exons using specific primers (Table S3) in two independent amplifications. IARC p53 database (http://www.p53.iarc.fr/) was consulted (15 January 2021) for p53 activity of mutants. A transcriptional activity < 75% was considered partially functional and classified as mutant [35].

### 2.9. ALDEFLUOR Assay

An ALDEFLUOR™ Kit (Stem Cell Technologies, Vancouver, BC, Canada) was used to detect enzyme ALDH1 activity in cultured cells according to the manufacturer’s instructions. After treatments, cells were incubated with BODIPY-aminoacetaldehyde (BAAA), a fluorescent non-toxic substrate for ALDH, which was converted into BODIPY-aminoacate (BAA) and retained inside the cells. Viable ALDH1+ cells were quantified by flow cytometry on a FACS Aria IIIu (BD Biosciences, San Jose, CA, USA). The specific inhibitor of ALDH, diethylaminobenzaldehyde (DEAB), was used to control for background fluorescence.

### 2.10. Isolation and Characterization of CSCs

Enrichment of CSCs was achieved via serial trypsin treatment, as previously described [36,37]. Briefly, cells at 60–80% of confluence were washed with PBS and treated with 0.05% trypsin for 2 min at 37 °C. Detached cells were plated and allowed to attach for 24 h. Then, the above procedure was repeated, and the cells obtained were considered CSCs. After the first trypsin treatment, remaining cells attached to the dishes were washed twice with PBS and incubated with 0.05% trypsin for 4 min at 37 °C. Cells detached from this trypsinization were discarded. Dishes with remaining trypsin-resistant cells were considered non-CSCs

Once isolated, cell surface marker levels of CSCs were determined with human anti-CD44-PE, anti-CD326-FITC, and anti-CD133-APC antibodies (Biolegend, San Diego, CA, USA). Samples were measured and analyzed by flow cytometry on a FACS Aria IIIu (BD Biosciences, San Jose, CA, USA).

### 2.11. Sphere Forming Assay

For self-renewal analysis, 24 h after transfection with either pCMV6-*HMOX1* or an empty vector, CSCs and non-CSCs cells were collected and quantified. Then, 3000 cells were resuspended in sphere culture medium (DMEM:F12, 1% penicilin/streptomycin, B27, 10 μg/mL ITS, 1 μg/mL Hydrocortisone, 4 ng/mL Heparin, 10 ng/mL EGF, and 20 ng/mL FGF) in ultra-low attachment 24-well plates (Corning). Spheres >75 μM diameter were counted after 4 days by light microscopy.

### 2.12. MTT Assay

Cells were seeded in 96 well-plates at a concentration of 40,000 cells/mL, allowed to attach overnight, and transfected with either pCMV6-HMOX1 or an empty vector. Then, the cells were treated with the vehicle or the corresponding drug, 24 h after transfection. After 72 h, 10 μL of 5 mg/mL MTT were added to each well. Four hours later, cells were lysed with 100 μL buffer (20% SDS in 50% formamide at pH 4.7) at 37 °C overnight. Absorbance was measured in a TRIAD Multimode Reader series, (Dynex Technologies, Chantilly, VA, USA) at 570 nm. Means were estimated from the results of four samples in each experimental group. All experiments were performed two times.

### 2.13. Statistical Analysis

For each patient, mRNA levels of genes in tumor samples were normalized to mRNA levels in normal mucosa. Descriptive statistics were reported as medians with interquartile ranges (IQR) for continuous variables and as whole numbers and percentages for categorical variables. Low and high levels of CSCs markers were obtained through the median of the mRNA expression levels in our cohort of patients. Associations between clinicopathological features of CRC patients and gene expression were analyzed with the Kruskal–Wallis and Mann–Whitney non-parametric tests. For the correlation analysis, the Pearson test was used after transforming the variables applying natural logarithms. All confidence intervals (CIs) were stated at the 95% level. Statistical significance was defined as *p* < 0.05. All statistical calculations were performed using SPSS software version 15.0 for Windows (IBM, Chicago, IL, USA).

## 3. Results

### 3.1. HO-1 Overexpression Induces Stemness in CRC In Vitro Regardless of p53 Status

**Figure 1:**
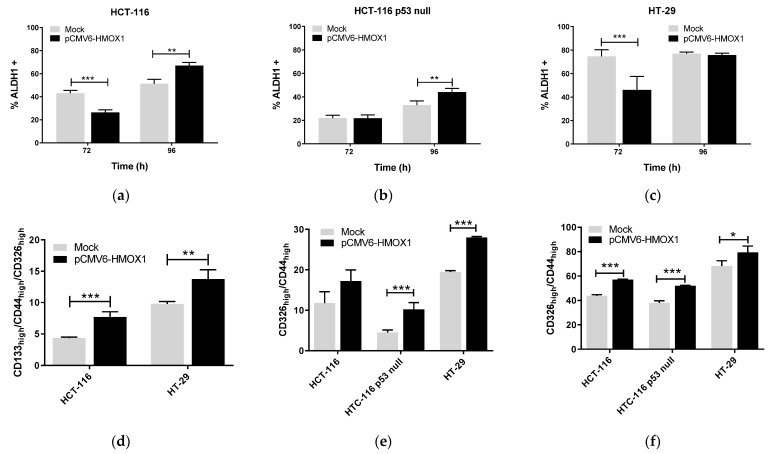
HO-1 overexpression increases stemness in CRC regardless of p53 status. Cells were pCMV6-*HMOX1* or mock transfected, collected at 72 and 96 hours after transfection, and used to characterize the percentage of ALDH1+ cells by flow cytometry in the total population (TP) of (**a**) HCT-116, (**b**) HCT-116 p53 null, and (**c**) HT-29 CRC cells. In other experiments, cells were collected at 96 h after pCMV6-*HMOX1* or empty vector (mock) transfections and used to quantify, in the TP, (**d**) the percentage of CD133_high_/CD44_high_/CD326_high_ and (**e**) the percentage of CD44_high_/CD326_high_ cells or (**f**) the percentage of CD44_high_/CD326_high_ cells in CSCs subpopulations, obtained as described in the Materials and Methods section. Data represent the mean ± SD of two experiments performed in duplicate. *, *p* < 0.05; **, *p* < 0.01; ***, *p* < 0.001.

In order to investigate whether HO-1 conferred stem cell properties on CRC cells, we used the pCMV6-*HMOX1* plasmid to transiently overexpress HO-1 in HCT-116, HCT-116 p53 null, and HT-29 cells. Overexpression of HO-1 increased aldehyde dehydrogenase 1 positive (ALDH1+) subpopulation, representing the subpopulation of CSCs, in HCT-116 and HCT-116 p53 null cells at 96 h after transfection (Figure 1a,b). In HT-29 cells, the percentage of ALDH1+ cells was lower in pCMV6-HMOX1 versus mock transfected cells at 72 h after transfection, whereas the percentage of this subpopulation was similar in control (mock) and HO-1 overexpressing cells at 96 h (Figure 1c).

The percentage of cells with high expression of CSCs markers in the total population (TP) of cells increased in pCMV6-*HMOX1* versus mock transfected cells at 96 h after transfection. At this time, the percentage of CD133_high_/CD44_high_/CD326_high_ cells significantly increased in the TP of pCMV6-*HMOX1* versus mock HCT-116 and HT29 transfected cells at 96 h after transfection (Figure 1d). We did not find triple-labeled subpopulation cells in the HCT-116 p53 null line; however, the percentage of CD44_high_/CD326_high_ cells significantly increased in the TP of pCMV6-*HMOX1* versus mock HCT-116 p53 null transfected cells at 96 h after transfection (Figure 1e). Similarly, in HT-29 cells, the percentage of the CD44_high_/CD326_high_ subpopulation significantly increased in the TP of pCMV6-*HMOX1* versus mock transfected cells at 96 h after transfection (Figure 1e). In addition, the percentage of CD44_high_/CD326_high_ cells increased in the CSCs subpopulation of pCMV6-*HMOX1* versus mock transfected cells at 96 h after transfection in the three cell lines studied (Figure 1f). These results indicate that HO-1 overexpression increases the proportion of cancer cells with a CSC phenotype in the different subpopulations analyzed.

**Figure 2:**
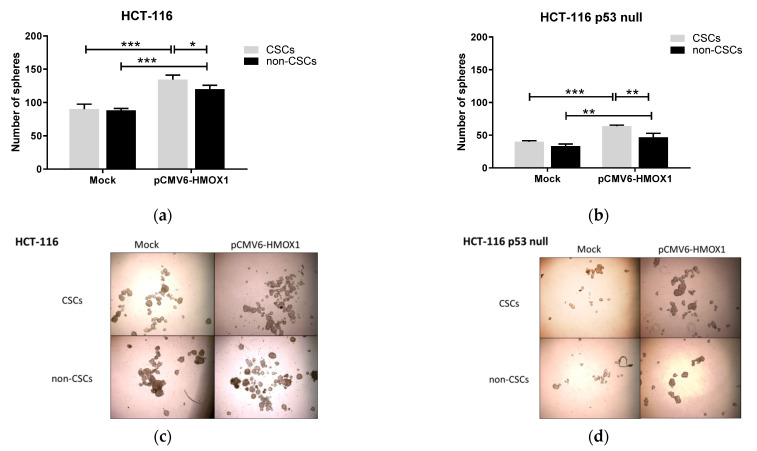
HO-1 overexpression increases self-renewal ability of isolated CRCssubpopulations in cultured cells, regardless of p53 status. The number of spheres formed by subpopulations obtained from mock and pCMV6-*HMOX1* (**a**) HCT-116 and (**b**) HCT-116 p53 null transiently transfected cells. Representative images of tumorospheres formed from different subpopulations of pCMV6-*HMOX1* (**c**) HCT-116 and (**d**) HCT-116 p53 null transiently transfected cells. TP: total population; CSCs: cancer stem cells subpopulation; non-CSCs: non-cancer stem cells subpopulation. *, *p* < 0.05; **, *p* < 0.01; ***, *p* < 0.001.

Next, we studied the anchorage-independent growth of CSCs and non-CSCs subpopulations extracted from HCT-116 and HCT-116 p53 null after transient overexpression of HO-1 under free-serum conditions. As shown in Figure 2, CSCs and non-CSCs from both cell lines formed spheres with similar efficiency in mock transfected cells; however, CSCs subpopulations formed spheres more efficiently than non-CSCs when parental cells were transfected with the pCMV6-*HMOX1* plasmid, indicating that HO-1 overexpression increases the self-renewal capacity of CSCs subpopulations, regardless of p53 status.

### 3.2. HO-1 Overexpression Induces ECE-1 Expression and ET-1 Production by CRC Cells

We next investigated the mechanism by which HO-1 could induce stemness in CRC. As mentioned before, HO-1 could modulate ET-1 production in several models of health and disease [37,38,39,40,41].

**Figure 3:**
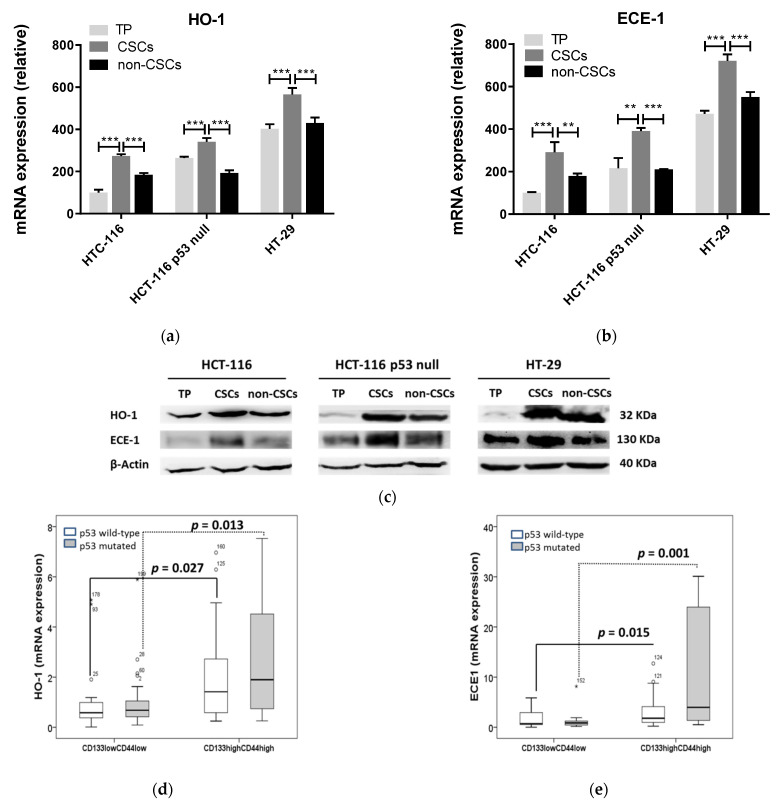
HO-1 and ECE-1 expression is higher in the CSCs vs. non-CSCs subpopulation in CRC regardless of p53 status. The CSCs and non-CSCs subpopulations from HCT-116, HT-116 p53 null, and HT-29 cells were obtained as described in the Materials and Methods section and used to measure mRNA expression of (**a**) HO-1 and (**b**) ECE-1. Data represent the relative expression of HO-1 and ECE-1 to the TP of HCT-116 and express the mean ± SD of two experiments performed in duplicate. **, *p* < 0.01; ***, *p* < 0.001. (**c**) Protein expression of HO-1 and ECE-1 in HCT-116, HT-116 p53 null, and HT-29 cells in the TP, CSCs, and non-CSCs subpopulations. β-actin was used as housekeeping. TP: total population; CSCs: cancer stem cells subpopulation; non-CSCs: non-cancer stem cells subpopulation. Box plots representing the relative mRNA expression of (**d**) HO-1 and (**e**) ECE-1 in CRC samples from patients considering the levels of CSCs markers and the status of p53. Data represent the median and the interquartile range of the genes analyzed. *, distant values more than 3 box lengths from 75th percentile; °, distant values more than 1.5 box lengths from 75th percentile.

It was interesting to find high mRNA and protein content of both HO-1 and ECE-1 in the CSC versus the non-CSCs subpopulation in the three cell lines studied (Figure 3a–c). We also analyzed the mRNA expression of HO-1, ECE-1, and the CSCs markers CD44 and CD133 by quantitative RT-PCR in 150 cases of CRC patients. Tumors showing high expression of CSCs markers (CD133_high_CD44_high_) also exhibited higher expression of HO-1 (Figure 3d) and ECE-1(Figure 3e) than tumors with low expression of these markers (CD133_low_CD44_low_). It was very interesting to find that HO-1 and ECE-1 expression highly correlated in both CD133_high_CD44_high_ and CD133_high_CD44_high_ tumors, while no correlations were found between HO-1 and END-1 (Table S3). The status of P53 did not influence any of the results mentioned above (Figure 3 and Table S3).

**Figure 4:**
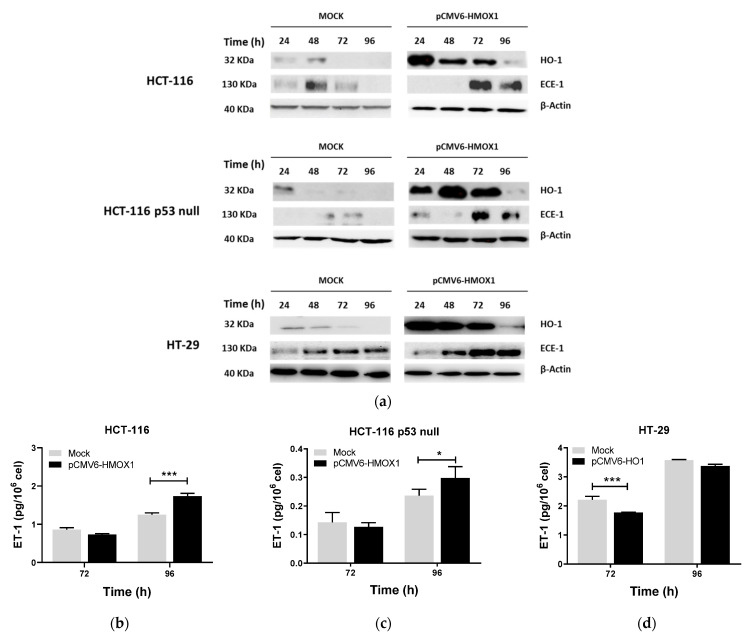

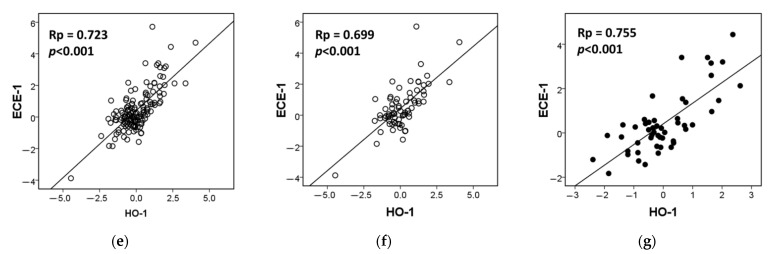
HO-1 overexpression induces ECE-1 expression and ET-1 production in CRC cells regardless of p53 status. Cells were transiently transfected with pCMV6-*HMOX1* or an empty vector and collected 24–96 h later for (**a**) HO-1 and ECE-1 protein expression by western blotting or ET-1 measurements in the supernatants of (**b**) HCT-116, (**c**) HCT-116 p53 null, (**d**) and HT-29 cells. Data represent the median ± SD of two experiments in duplicate. *, *p* < 0.05; ***, *p* < 0.001. Correlation of ECE-1 and HO-1 expression in tumors from CRC patients considering (**e**) all cases, (**f**) p53 wild-type tumors, and (**g**) p53 mutated tumors.

Next, we studied whether HO-1 could regulate ET-1 production in CRC. As shown in Figure 4a, HO-1 induced ECE-1 expression at 72 and 96 h after transfection with the pCMV6-*HMOX1* plasmid in HCT-116, HCT-116 p53 null, and HT-29 cells versus vector (mock) transfected cells. In addition, HO-1 overexpression induced ET-1 synthesis in HCT-116 and HCT-116 p53 null cells at 96 h after transfection (Figure 4b,c). In HT-29 cells, HO-1 overexpression induced inhibition of ET-1 at 72 h, whereas, at 96 h, its levels increased until reaching the levels of control (mock) cells (Figure 4d).

In the cohort of CRC patients, HO-1 and ECE-1 expressions positively correlated in all cases (Rp = 0.723, *p* < 0.001; Figure 4e) in tumors harboring a wild-type p53 (Rp = 0.699, *p* < 0.001; Figure 4f) and in tumors with mutations in this gene (Rp = 0.755, *p* < 0.001; Figure 4g). END-1, the first gene implicated in the synthesis of ET-1, did not correlate with HO-1 at any of the conditions mentioned above (Rp = 0.042, *p* = 0.614; Rp = 0.042, *p* = 0.905; Rp = 0.093, *p* = 0.506, for all cases, p53 wild-type tumors, and p53 mutated tumors, respectively). As shown in Table S2, none of these genes correlated with any of the clinicopathological features of the patients included in the study.

**Figure 5:**
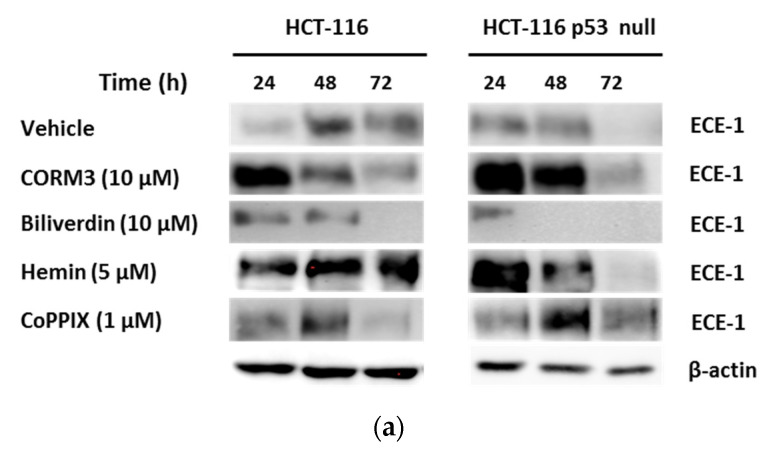

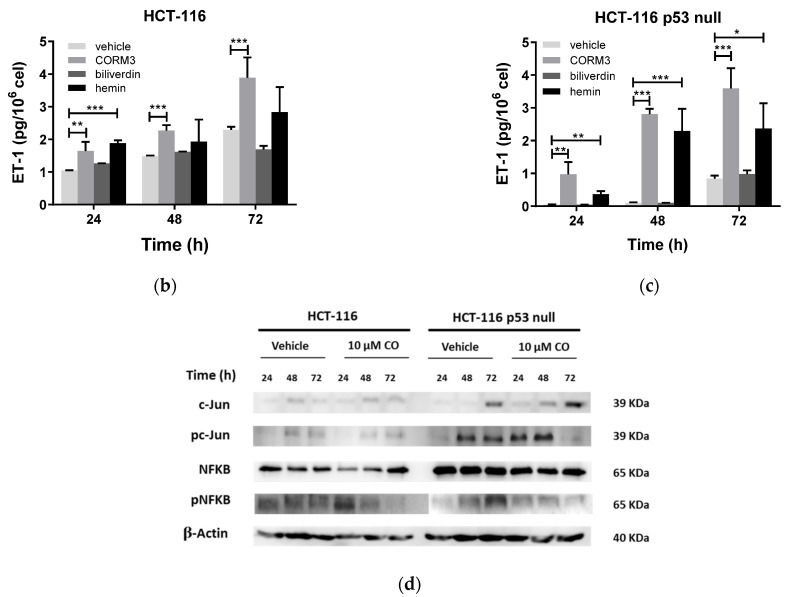
CO induces ECE-1 expression in CRC cells in vitro through the activation of NF-kβ and c-Jun in HCT-116 and HCT-116 p53 null cells, respectively. HCT-116 and HCT-116 p53 null cells were treated with 10 µM CORM3, 10 µM biliverdin, 5 µM hemin, and 1 µM CoPPIX during 24, 48, and 72 h. After treatments, we collected (**a**) cells for the analysis of ECE-1 protein expression by western blotting and supernatants for the analysis of ET-1 in (**b**) HCT-116 and (**c**) HCT-116 p53 null cells. Data represent the median ± SD of two experiments performed in duplicate. *, *p* < 0.05; **, *p* < 0.01; ***, *p* < 0.001. (**d**) The mechanism of CO-induced ECE-1 expression in CRC cells. Cells were treated with 10 µM CORM3 and collected for the analysis of c-Jun, pc-Jun, NF-kβ, and pNF-kβ proteins expression in whole cell lysates by western blotting. The expression of β-actin was used as housekeeping.

We next investigated which of the subproducts of the HO-1 reaction is responsible for ECE-1 activation after HO-1 overexpression in CRC cells. To assess this, we treated cells with 10 µM CORM3 (a CO donor) and 10 µM biliverdin. We also used 5 µM hemin and 1 µM CoPPIX treatments as positive controls. CORM3, hemin, and CoPPIX induced ECE-1 protein expression at the doses used in both HCT-116 and HCT-116 p53 null cells, whereas biliverdin was unable to induce it (Figure 5a). We also studied ET-1 production by cells after treatments at the doses specified above (Figure 5b,c). CORM3 induced a significant increase in ET-1 production at all times analyzed in both cell lines. Treatment with hemin induced increased ET-1 production in HCT-116 cells, although only after 24 h, while this effect appeared after 24, 48, and 72 h in HCT-116 p53 null cells. After showing that CO is responsible for the activation of ECE-1, we next investigated the mechanism by which it carries out this effect. As shown in Figure 5d, CO seemed to induce ECE-1 expression through the activation of pNF-kβ and pc-Jun in HCT-116 and HCT-116 p53 null cells, respectively.

### 3.3. HO-1 Overexpression Induces Stemness in CRC Cell Lines through ECE-1/ET-1 Only in p53 Wild-Type Cells

**Figure 6:**
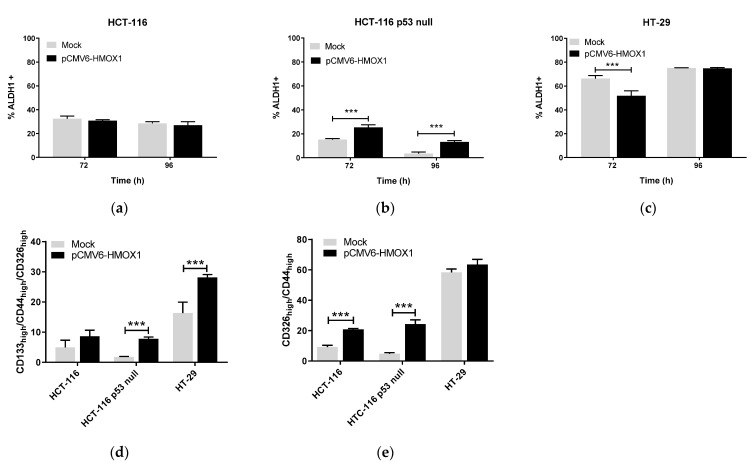
Blockade of endothelin-1 receptors eliminates the HO-1-induced stemness only in p53 wild-type CRC cells. Cells were pCMV6-*HMOX1* or mock transfected, treated with 10 μM bosentan 24 h after, collected at 72 and 96 h after transfection, and used to characterize the percentage of ALDH1+ cells by flow cytometry in the TP of (**a**) HCT-116, (**b**) HCT-116 p53 null, and (**c**) HT-29 CRC cells. In other experiments, cells were collected at 96 h after pCMV6-*HMOX1* or empty vector (mock) transfections and used to quantify, in the TP, (**d**) the percentage of CD133_high_/CD44_high_/CD326_high_ and (**e**) CD44_high_/CD326_high_ cells. Data represent the mean ± SD of two experiments performed in duplicate. ***, *p* < 0.001.

To study whether HO-1 overexpression induces stemness in our model of CRC in vitro through ET-1 produced by ECE-1, we analyzed the percentage of ALDH1+ cells after HO-1 overexpression in the presence of bosentan, an antagonist of both ETRA and ETRB receptors. In this case, and contrary to what happened in the absence of bosentan, HO-1 overexpression was not able to induce an increase in the ALDH1+ subpopulation in HCT-116 cells (Figure 6a), and the percentage of CD133_high_/CD44_high_/CD326_high_ cells in the TP was similar in pCMV6-*HMOX1* versus mock transfected cells (Figure 6d). However, the percentage of CD44_high_/CD326_high_ cells increased in pCMV6-*HMOX1* versus mock transfected cells (Figure 6e). Therefore, HO-1 induces stemness in CRC cells harboring a wild-type p53 trough ET-1.

On the contrary, the addition of bosentan after HO-1 overexpression led to an increase in the percentage of ALDH1+ cells at 72 and 96 h after transfection (Figure 6b) as well as to the appearance of the CD133_high_/CD44_high_/CD326_high_ subpopulation in HCT-116 p53 null cells (Figure 6e). In HT-29 cells, the presence of bosentan after HO-1 overexpression did not modify the percentage of ALDH1+ cells, although the CD44_high_/CD326_high_ subpopulation seemed to decrease, while the difference in the percentage of the CD133_high_/CD44_high_/CD326_high_ subpopulation increased in pCMV6-*HMOX1* versus mock transfected cells (Figure 6d,e), when comparing this result in the absence of bosentan (Figure 1d). According to these results, the blockade of ET-1 receptors induces an increase in the CSCs subpopulation in cells without an active p53 or with a mutated p53 with a gain of function, and ET-1 seems to act as a protective agent in these cells after HO-1 overexpression.

**Figure 7:**
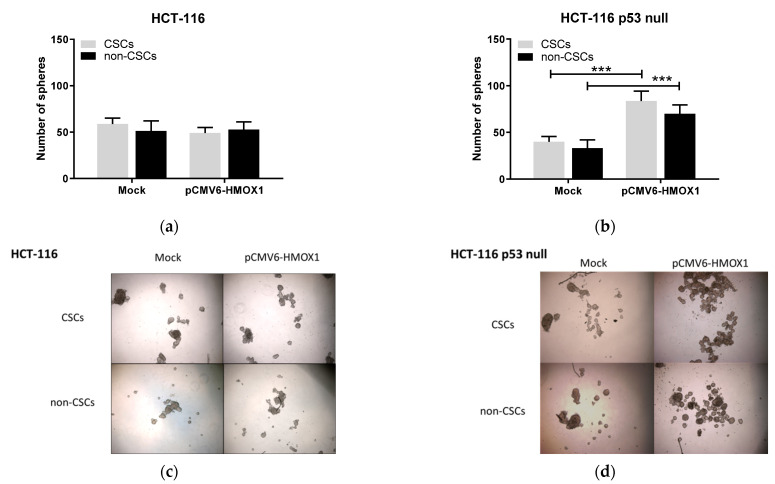
Blockade of endothelin-1 receptors eliminates the HO-1-induced self-renewal ability in p53 wild-type CRC cells and enhances it in p53 null cells. The number of spheres formed by subpopulations obtained from mock and pCMV6-*HMOX1* (**a**) HCT-116 and (**b**) HCT-116 p53 null transiently transfected cells. Representative images of tumorospheres formed from different subpopulations of pCMV6-*HMOX1* (**c**) HCT-116 and (**d**) HCT-116 p53 null transiently transfected cells. ***, *p* < 0.001.

The presence of bosentan in the cultured media also influenced the self-renewal capacity of CSCs and non-CSCs subpopulations extracted from transiently transfected HCT-116 and HCT-116 p53 null cells with empty vector (mock) and pCMV6-*HMOX1* plasmid. On these working conditions, the capacity of subpopulations to form spheres was similar in mock and pCMV6-*HMOX1* transfected HCT-116 cells (Figure 7a). However, CSCs and non-CSCs subpopulations from HCT-116 p53 null cells increased their ability to form spheres (Figure 7b). Considering these results, ET-1 increases the self-renewal capacity in cells with a wild-type p53 and decreases it in cells with an inactive p53 after HO-1 overexpression, and the presence of bosentan eliminates these effects.

### 3.4. HO-1 Overexpression Induces Resistance to 5-FU Treatment in CRC In Vitro

**Figure 8:**
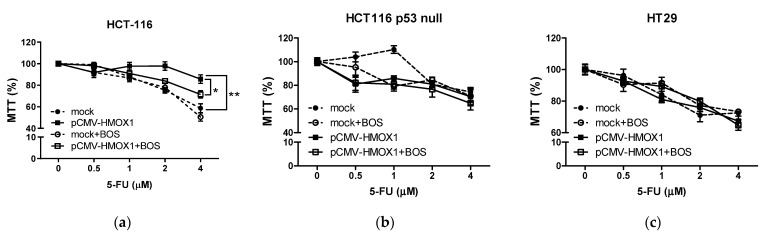
Influence of HO-1 overexpression on therapy resistance in CRC. As described in the Materials and Methods section, cells were seeded in 96 well-plates, transfected with vector or pCMV6-*HMOX1* plasmid, and treated with increasing doses of 5-FU (0–4 Μm) during 72 h in the presence or absence of 10 μM bosentan. The viability of cells was analyzed using the MTT assay in (**a**) HCT-116, (**b**) HCT-116 p53 null, and (**c**) HT-29 cells. Data represent the mean ± SD of two experiments performed in quadruplicate. *, *p* < 0.05; **, *p* < 0.01.

Given the implication of CSCs on therapy resistance in cancer, we analyzed whether HO-1 overexpression induced this effect on CRC in vitro and the mediation of the ECE-1/ET-1 system on it. The treatment of mock and pCMV6-*HMOX1* HCT-116, HCT-116 p53 null, and HT-29 transfected cells with 5-fluorouracil (5-FU) at different doses (0–4 μM) showed that HO-1 overexpression induced resistance to this treatment in HCT-116 cells (Figure 8a). We did not find different 5-FU sensitivity after HO-1 overexpression in HCT-116 p53 null nor in HT-29 cells (Figure 8b,c), at least at the doses of 5-FU used. The presence of bosentan in the culture media sensitized HCT-116 cells overexpressing HO-1 to 5-FU (Figure 8a); however, no effect was found in HCT-116 p53 null cells (Figure 8b). On the contrary, in HT-29 cells transfected with pCMV6-*HMOX1*, the presence of bosentan induced resistance to 5-FU (Figure 8c). We could conclude that HO-1 overexpression induces 5-FU resistance in cells with a wild-type p53 and that ET-1 is responsible, at least in part, for this effect. On the contrary, in cells with a non-wild-type p53, HO-1 overexpression seemed to not affect the response to 5-FU treatment at the doses used in this study. In addition, ET-1 increases the response to this treatment in cells with a mutated p53 with a gain of function.

## 4. Discussion

In the recent years, numerous research works have tried to elucidate the role of HO-1 in colorectal cancer. This has not been an easy task, since this enzyme is found in both cancer and stromal cells, including immune cells [38,39,40,41]. In addition, HO-1 in CRC cells is able to regulate immune-mediated cytotoxicity against CRC cells [18]. In this complex scenario, the results from the scientific literature have shown contradictory effects of HO-1 in this type of cancer [15,16,17,18,19,20], which highlights the need to continue this field of research. The metabolic status of cancer cells influences how heme-degrading enzymes modulate tumor growth. Conversely, CO and biliverdin can modulate lipid and glucose metabolism [42]. Specifically, CO can promote increased mitochondrial biogenesis and induce an anti-Warburg effect [43], which has been linked to a lower rate of proliferation and cell differentiation [44]. In addition, the presence of HO-1 in cytosol or its translocation into the nucleus can also affect its activity and, therefore, its effects on tumors [42].

We used the CRC cell lines HCT-116, HCT-116 p53 null, and HT-29 to conduct our in vitro model. These cells have the ability to form tumorospheres within 3–5 days after being seeded in appropriate plates and medium [45,46,47], which allowed us to carry out our study even though the transformation of the parental cells was performed by transient transfection [48,49].

In this study, we found that HO-1 regulates the proportion and phenotype of CSCs in CRC, a result that is not surprising considering that we also found a higher expression of this protein in the CSCs subpopulation compared to non-CSCs in cell cultures. In tumors from CRC patients, samples with a higher expression of CSCs markers also showed a higher expression of HO-1. These results agree with previous works that reported a direct implication of HO-1 in regulating CSCs in leukemia, glioma, melanoma, breast, pancreatic, and lung cancers [24,49,50,51]. According to our results, HO-1 could regulate stemness in CRC independently of p53; however, previous reports have demonstrated an important relationship between them [16,52]. In fact, HO-1 is a p53-dependent target gene that is responsible for p53-dependent cellular survival after oxidative treatment [52]. Other works have suggested that HO activity is involved in the regulation of p53 expression in normal [53] and cancer cells [54].

In our in vitro model, CO produced after HO-1 overexpression led to increased expression of ECE-1 and ET-1 secretion by CRC cells through the activation of NF-κB and AP-1 in cells harboring a wild-type p53 and in cells with a mutated p53, respectively. These transcription factors are essential for ECE-1 activation in endothelial cells [55]. The CO releasing molecule CORM3 has been reported to activate AP-1 in vitro [56,57]. The regulation of NF-κB by CO has been previously described in several models of disease [58,59]. This molecule is also able to induce ROS production in vitro [60]. Interestingly, ROS production and NF-κB activation are critical events in CSCs appearance and CRC initiation [61]. Biliverdin was unable to activate the ECE-1/ET-1 pathway in our study. Biliverdin is converted to bilirubin by bilirubin reductase [62]. The ROS scavenging properties of both heme metabolites contribute to their antitumoral activities [11,63]. We did not test the possible role of ferrous ions on ECE/ET-1 induction by HO-1 overexpression. Similarly to bilirubin, literature to date has shown protective effects of ferrous iron against cancer, mainly due to the induction of ferroptosis and cell death [11,41,64,65].

The endothelin system regulates stem properties of cells in CRC. The END-1 gene is highly expressed in CSCs isolated from cultured CRC cells [66]. ET-1 regulates important pathways for the maintenance of the stemness phenotype, such as MAPK, PI3K/Akt, and Wnt/β-catenin [29]. However, given the short half-life of ET-1, its biological effects are totally dependent on the maintenance of a critical concentration by ECE-1 [67]. Of the four described isoforms of ECE-1 (a, b, c and d), ECE-1c has been implicated in CSCs generation in CRC [67]. In our study, we did not analyze which isoform of ECE-1 increased after HO-1 overexpression. However, ETRA and ETRB receptors blockaded with bosentan led to a decrease in the ALDH1+ population, in the percentage of CD133_high_/CD44_high_/CD326_high_ cells, and in the resistance to 5-FU treatment in the total population and a decrease in the ability for self-renewal of the CSCs subpopulation of wild-type p53 cells. Previous studies have reported that ETRA receptors are responsible for ET-1-mediated stemness in CRC [29].

On the other hand, treatment with bosentan after HO-1 overexpression induced an increase in the ALDH1+ and the appearance of CD133_high_/CD44_high_/CD326_high_ subpopulations in the total population, and it increased the ability to form spheres in the CSCs subpopulation of cells lacking p53. Similarly, in cells harboring a mutated p53, the ETR blockade induced a loss of CD44_high_/CD326_high_ cells and an increase in CD133_high_/CD44_high_/CD326_high_ ones. These results could indicate that even though HO-1 overexpression induces increased expression of ECE-1 and ET-1 production regardless of p53, in cells without an active p53 or even a p53 with gain of function, like HT-29 cells, another mechanism that does not involve the ECE-1/ET-1 system is responsible for the increase in the stem subpopulation. More interestingly, the HO-1-induced ECE-1/ET-1 system seems to exert a protective effect in these p53 not-active or with gain of function CRC cells. Contrary to these results, previous reports have shown that treatment with bosentan is proapoptotic and antiproliferative in HT-29 [68] cells and sensitizes them to Fas-L-induced cell death [69,70]. An ET-1-independent mechanism of ECE-1 in CRC and other cancers has also been described [67], but in these cases it acts as a protumoral protein [67]. On the contrary, clinical trials with approved endothelin receptor antagonists, including bosentan, were not so promising, with no statistically significant results, even though the drugs were well-tolerated [71]. Although more research is warranted to confirm our results, HO-1 overexpression probably activates or inactivates certain unknown pathways that could induce these contrary results after treatment with bosentan in our cell model.

## 5. Conclusions

HO-1 regulates stemness and resistance to 5-FU treatment in CRC in vitro. CO produced after HO-1 overexpression seemed to activate the ECE-1/ET-1 pathway, which could be the final effectors of HO-1 protumoral events in p53 wild-type cells. Surprisingly, in cells with a non-active p53 or a gain-of-function mutated p53, ECE-1-produced ET-1 seems to act as a protective molecule. These results could implicate that patients carrying tumors with high expression of both HO-1 and ECE-1 and a non-wild-type p53 should be considered for HO-1 based-therapies instead of ET-1 antagonists-based ones.

## Supplementary Materials

The following are available online at https://www.mdpi.com/article/10.3390/jpm11060509/s1, Table S1: Characteristics of the patients included in the study; Table S2: Relationship between HO-1 and ECE-1 expression with the clinicopathological characteristics of the patients included in the study; Table S3: Correlations between HO-1 and genes related to ET-1 synthesis, according to p53 status and levels of CSC markers.
