# Supplementary material for: Endothelin-1 as a Mediator of Heme Oxygenase-1-Induced Stemness in Colorectal Cancer: Influence of p53
Source: J Pers Med. 2021 Jun 4;11(6):509. doi: 10.3390/jpm11060509 (PMC8227293; doi:10.3390/jpm11060509)
Supplement: Supplementary file 1 [file jpm-11-00509-s001.zip › jpm-1227179-supplementary.pdf]

Table S1. Characteristics of the patients included in the study

Characteristic	N (%)
**Age**	
≤72	78 (52.0)
>72	72 (48.0)
**Gender**	
Female	92 (61.3)
Male	58 (37.7)
**Location**	
Colon	141 (94)
Rectum	9 (6)
**Histological type**	
Well differentiated	38 (25.3)
Moderately differentiated	94 (62.7)
Poorly differentiated	18 (12.0)
**pTMN Stage**	
Stage I	15 (10.0)
Stage II	59 (39.3)
Stage III	64 (42.7)
Stage IV	12 (8.0)

Table S2. Relationship between HO-1 and ECE-1 expression with the clinicopathological characteristics of the patients included in the study.

	P53 wild-type					
	**HO-1**	**P[a]**	**ECE-1**	**P[a]**	**EDN-1**	**P**
**Age[b]**						
≤ 72	0.78 (0.46-2.68)	ns	1.18 (0.80-3.81)	ns	0.85 (0.52-1.21)	ns
>72	1.14 (0.64-2.00)		1.11 (0.65-2.83)		0.80 (0.26-1.47)	
**Gender**						
Male	0.90 (0.54-2.47)	ns	1.16 (0.85-4.91)	ns	1.14 (0.65-3.55)	ns
Female	0.74 (0.52-1.70)		1.15 (0.62-2.27)		1.32 (0.47-2.72)	
**TNM stage**						
I+II	0.90 (0.52-2.06)	ns	1.17 (0.77-2.53)	ns	0.86 (0.40-1.21)	ns
III+IV	0.74 (0.53-2.90)		1.11 (0.63-8.06)		0.84 (0.42-1.38)	
**Differentiation grade**						
Well	0.58 (0.45-1.23)	ns	1.04 (0.77-2.19)	ns	0.66 (0.42-1.21)	ns
Moderately	1.14 (0.58-2.94)		1.34 (0.70-4.51)		0.90 (0.38-1.51)	
Poor	1.14 (0.71-3.52)		1.40 (1.06-3.79)		0.74 (0.31-1.08)	
	**P53 mutated**					
	**HO-1**	**P[a]**	**ECE-1**	**P[a]**	**EDN-1**	**P[a]**
**Age[b]**						
≤ 72	1.18 (0.80-3.81)	ns	1.11 (0.46-5.26)	ns	0.85 (0.39-2.15)	ns
>72	1.11 (0.65-2.83)		1.25 (0.63-2.10)		0.92 (0.48-1.77)	
**Gender**						
Male	1.16 (0.85-4.91)	ns	1.14 (0.65-3.55)	ns	0.90 (0.55-2.15)	ns
Female	1.15 (0.62-2.27)		1.32 (0.47-2.72)		0.86 (0.30-1.67)	
**pTNM stage**						
I+II	1.17 (0.77-2.53)	ns	1.40 (0.64-2.83)	ns	0.86 (0.40-2.10)	ns
III+IV	1.11 (0.63-8.06)		1.02 (0.53-3.13)		0.89 (0.50-1.70)	
**Differentiation grade**						
Well	1.04 (0.77-2.19)	ns	1.36 (0.64-2.31)	ns	0.72 (0.31-2.37)	ns
Moderately	1.34 (0.70-4.51)		1.25 (0.52-4.66)		0.95 (0.64-1.74)	
Poor	1.40 (1.06-3.79)		1.39 (0.70-0.85)		0.72 (0.32-1.33)	

[a]P values were obtained using the Mann-Whitney or Kruskal-Wallis non-parametric tests; [b]Dicotomized by the median.

Table S3. Correlations between HO-1 and genes related to ET-1 synthesis according to p53 status and levels of CSC markers

	**P53 wild-type**	
	**$CD133_{low}CD44_{low}$**	**$CD133_{high}CD44_{high}$**
**ECE-1**		
[a]Rs	**0.700**	**0.604**
P	**0.001**	**0.001**
**EDN-1**		
[a]Rs	-0.346	-0.088
P	ns	ns
	**P53 mutated**	
	**$CD133_{low}CD44_{low}$**	**$CD133_{high}CD44_{high}$**
**ECE-1**		
[a]Rs	**0.648**	**0.661**
P	**0.001**	**0.037**
**EDN-1**		
[a]Rs	0.142	0.322
P	ns	ns

[a]Pearson's correlation coefficient
